# Acute Rheumatism in the Young

**Published:** 1906-11-10

**Authors:** 


### SPECIAL ARTICLE.

### ACUTE RHEUMATISM IN THE YOUNG.

Little doubt remains that acute rheumatism is an infective disease, although the identity of the organism has not been conclusively established. The researches of Poynton and Paine point to the operation of a diplococcus which has been detected in valvular vegetations and other rheumatic lesions, and which is stated to be able to produce a multiple arthritis with endocardial lesions upon inoculation into animals. Whether this particular organism is the specific cause of the disease, or whether the latter depends upon a diathetical susceptibility to the invasion of the blood-stream by streptococci of various sorts, as some authorities assert, is a matter of secondary clinical importance. It is unquestionable that hereditary influences play an important part in the incidence of the malady, and it is almost equally certain that the disease is microbic in origin.

Clinically considered the manifestations of acute rheumatism in childhood and in adult life respectively are so different that, but for the cardiac associations which are common to both, the unity of the two might well come into question. So much is this the case that until a comparatively recent date acute rheumatism was held to be relatively a rarity in childhood. For example, of 655 cases analysed by Whipham 80 per cent. were found to occur between the ages of 20 and 40 years. There can be little doubt that these figures do not represent a general truth. Indeed they do but illustrate the width of the breach between the childish and the adult types of the disease. Specific bacteriology apart, we have to conceive of acute rheumatism as a general microbic infection of the blood-stream—that is to say, a septicæmia. This conclusion is enforced by the frequent occurrence of vegetative valvulitis. We know that the valvular endocardium is liable on occasions to be attacked by a number of organisms whose identity is better established than that of the organism or organisms responsible for rheumatic fever, such, for instance, as the pneumococcus, the gonococcus, the influenza bacillus, and others. In these cases, which are known as infective or malignant endocarditis, it is generally possible to cultivate the causative organism from the blood-stream during life, a proceeding which establishes definitively the septicæmic nature of the disease. We may conclude, therefore, on the score of analogy, that in the case of acute rheumatism we have to deal with a microbic infection which very frequently at all events becomes a septicæmia, as frequently, that is to say, as the disease attacks the heart. But the rheumatic infection differs in one important clinical detail from that of the "malignant" varieties of endocarditis. Whereas in the latter instances recovery is a matter of great rarity when once the diagnosis has been established, in the case of rheumatism it is the rule. We must recognise therefore that the rheumatic infection, however grave its legacies, is one of a relatively low virulence.

Acute rheumatism as defined by Osler is "an acute non-contagious fever, dependent upon an unknown infective agent, and characterised by multiple arthritis and a marked tendency to inflammation of the fibrous tissues." This is a much juster definition of the adult than of the childish disease. Everyone is familiar with the dramatic features of adult rheumatic fever, the suddenness of its onset, the high fever, and the extreme urgency of the articular symptoms. Upon this picture we do not propose to dwell, but rather upon the unobtrusive and insidious malady which marks out so many children in this country for a shortened life of more or less chronic invalidism.

We have said that Osler's definition is hardly applicable to the rheumatic fever of juveniles, for at this early period of life both the fever and the arthritis characteristic of the adult disease assume a degree of prominence quite insignificant in comparison with the true gravity of the affection. It would be more correct to define this malady of youth as "a subacute infective disease, chiefly affecting children between the ages of three and ten years, and characterised by a low degree of fever, some stiffness of the extremities or some slight articular pain, and by a great tendency towards the development of valvular endocarditis." But in point of practice it is no rarity to meet with children the subjects of valvular disease which is almost certainly rheumatic in origin in the case of whom it is nevertheless impossible to obtain any history of articular manifestations whatever.

If, however, a type be demanded, it may be instanced as follows:—A child of, say, five years is brought for medical advice on account of vague pains about the extremities, or, quite commonly, for mere malaise and listlessness of indefinite duration but extending over a period of several weeks. Upon examination it is discovered that there is a moderate degree of fever, say to 100° F., that the tongue is slightly coated, and that the apex beat of the heart is slightly displaced outwards. At the same time the rate of the heart-beat is slightly accelerated, and the general appearance of the patient is anæmic. Auscultation of the heart reveals a soft, blowing systolic murmur at the apex, and often a reduplication of the second sound in this region. At this stage it is impossible to say whether the murmurous heart-sound depends upon some slight degree of cardiac dilatation, or upon the anæmia, or whether again it is the evidence of organic valvular disease. If now, suspicion being aroused, close inquiry be made into the antecedent history, it may be remarked by the parent, but incidentally and as a matter of minor interest, that the child has limped a little, or complained of stiffness, or of breathlessness upon exertion. Further, it may come out upon questioning that the child is subject to sore throats, or that some other member of the family has been affected with rheumatic fever or chorea. If such a child be now kept under observation (assuming that the probabilities of the case are fulfilled, and that rheumatism is indeed responsible for the symptoms), the following sequence of events may be expected. The pains and fever are rapidly dispelled by the use of salicylate therapeutics, and the constitutional condition of the patient becomes practically normal. None the less the ambiguous heart-sounds undergo a gradual evolution towards the development of a murmur or murmurs undeniably organic. The systolic murmur heard at the apex becomes more defined and louder, assuming at the same time the quality of conduction toward the axilla, which in an accepted characteristic of an organic mitral murmur. At the same time the true significance of the reduplicated second sound may manifest itself in the development of a definite murmur in diastole, and we know that thenceforward that heart is handicapped for good.

Some such a picture may be taken as representative of the prevailing type of juvenile rheumatism. The point for emphasis is the subordinate prominence of fever and articular symptoms, and the paramountcy of heart lesions. Other valves than the mitral may, of course, share in the assault of the disease, while the pericardium is particularly prone to be simultaneously affected. On occasion this latter structure bears the brunt of the attack. When this is the case the clinical picture loses some of its ambiguity, for when the pericardium is acutely inflamed there is almost always some complaint of præcordial pain, a symptom quite commonly lacking when the heart-valves alone are affected. Precise diagnosis in the early stages of pericarditis is often no easy matter, for although the rough to and fro murmur characteristic of this lesion is frequently to be heard, the sounds are sometimes of so soft a quality as to make the distinction between endocarditis and pericarditis a very ill-defined one.

A common incident of juvenile rheumatism is the development of "rheumatic nodules." These are tumours of varying size, but mostly small—that is to say, the size of a split pea, situated along the sheaths of tendons or in the fibrous tissue coverings of bony prominences, such as the knees or elbows. Although these are the commonest situations, the fibrous nodules are often to be found over the clavicles, scapulæ, and ribs, and also occasionally in the scalp, where they may attain much larger dimensions. Structurally these rheumatic nodules consist of a loose connective tissue. They appear in crops, and may persist for several weeks, and, although their precise causation and relation to the disease are not certainly known, they must be regarded as evidence of activity of the infection, and are seldom, if ever, found without co-existing evidence of valvular involvement.

As in its onset, so in its course, juvenile rheumatism shows wide departures from the adult type of the disease. Thus fever of more than a moderate degree is quite uncommon, and hyperpyrexia an extreme rarity, while conformably with the milder type of the infection, mental disturbance in the nature of delirium is very infrequent. Erythematous eruptions, however, especially erythema marginatum, appear with an approximately equal frequency at all ages. The profuse perspirations of the adult disease are seldom seen in childhood. In a considerable proportion of cases acute rheumatism in childhood is associated with chorea. The exact relationship of these two diseases is still to some extent a moot point, but that there is often some very close association hardly admits of doubt. In families exhibiting the rheumatic diathesis chorea and rheumatism often appear to be interchangeable manifestations, some members being attacked by the one and some by the other. Further, a large number of rheumatic children at some time or another suffer from chorea, whether before, during, or after the rheumatic seizure.

It is easy to appreciate, in view of the potential gravity of the disease, the high importance of identifying it early. It is equally easy to understand how liable to oversight is a malady of so insidious a march. Unhappily, by the time that medical opinions are sought for the relief of rheumatic children the endocardium has too often already suffered assault. This misfortune, however, does not qualify the need for early diagnosis. As in all diseases the essential element in early diagnosis is suspicion. If it be borne in mind that a slight degree of muscular stiffness, such as may well be disguised under the term "growing-pains," or tonsillitis, or even mere listlessness and anæmia, may be the only outward signals of the rheumatic infection, the physician is not likely to sin in omission, however much parental ignorance may err. On the rare occasions when the disease assumes the acute articular form, diagnosis may for a while be very much in doubt. As an illustration of this uncommon type the following case may be cited. A boy of eight years came under notice for pain in his left hip. It was said that after a period of general malaise lasting two or three days the boy had been suddenly seized with a pain in his hip, so severe as to make standing impossible. He had always been healthy, but a sister had suffered from rheumatic fever. He was febrile, and complained bitterly of pain in his hip, but in no other joint. The heart appeared to be intact, and the considerable degree of constitutional disturbance prompted the suspicion that some acute infection in the nature of osteo-myelitis might be responsible for the symptoms. During the night, and indeed for five days, he was delirious and noisy, but the diagnosis was set at rest on the second day by the appearance of a painful swelling of the right wrist, accompanied by a profuse perspiration.

The immediate prognosis of juvenile rheumatism is good, although from time to time cases occur of children carried off by the virulence of the infection. In such cases active endocarditis and pericarditis generally combine to induce the fatal result. But far more often the attack passes off, leaving the prognosis to be determined by the presence and gravity of heart-lesions. Of all the incidental lesions affecting the heart pericarditis is that to which the most ominous significance must be attached. The importance of this inflammation lies in the fact that one of its most common legacies is a close adhesion between the two layers of the pericardium. Such an adhesive pericarditis throws upon the heart a burden which too often becomes after a few years intolerable and ends in heart-failure. It is a fact that the commonest single cause of death among children between the ages of eight and fourteen years is heart-failure resulting from rheumatic pericarditis, and that in a large majority of such cases adhesive pericarditis may be demonstrated in the deadhouse. The general prognosis of valvular disease of the heart is too large a subject to be dealt with in this review.

The most important therapeutic consideration is, beyond all comparison, rest. It is a matter of general experience that the more frank and acute an attack of rheumatism the less is the liability to serious cardiac lesions, and the most reasonable explanation of the fact is that the urgency of the symptoms in such a case commands early and complete rest. It is the physician's business to replace the insistence of symptoms when these are mitigated. It is not too much to say that complete recumbency for a period of three months at least should be enjoined upon any child affected with rheumatism. In this way only can we hope to abate the lingering disabilities of rheumatic children. Salicylates have, of course, their place in the therapeutic management of the disease, but it is certain that the relief they afford is often a curse rather than a blessing, since it reduces the obviousness of the call for rest. Finally, it should be said that children of a stock known to be liable to rheumatism should be protected during childhood from exposure to cold. The swimming-bath has much to answer for.

